# Spontaneous Retroperitoneal Hematoma: A Rare Presentation of Polyarteritis Nodosa

**DOI:** 10.1177/2324709619858120

**Published:** 2019-06-24

**Authors:** Asad Ullah, Asghar Marwat, Krithika Suresh, Ahmed Khalil, Saba Waseem

**Affiliations:** 1 Conemaugh Memorial Medical Center, Johnstown, PA, USA

**Keywords:** retroperitoneal hematoma, polyarteritis nodosa

## Abstract

Spontaneous retroperitoneal hematoma is a rare clinical entity that is most commonly caused by renal tumors and vascular disease. In this article, we present a case of spontaneous retroperitoneal hemorrhage caused by polyarteritis nodosa in a patient who presented with severe left flank pain. He underwent computed tomography angiography of his abdomen that showed left retroperitoneal hematoma, which was followed by arteriogram that showed multiple bilateral renal artery aneurysms with active extravasation, findings consistent with polyarteritis nodosa. The patient underwent successful coiling of the bleeding vessel that secured the bleeding and was started on high-dose prednisone, which resulted in resolution of his symptoms.

## Case

Our patient is a 67-year-old gentleman with past medical history significant for hypertension, diabetes mellitus type 2, and chronic obstructive pulmonary disease, who presented to our emergency department with sudden onset of severe left flank pain. His pain started while he was watching TV at his home and was progressively getting worse, which prompted him to come to the emergency department for further evaluation. He denied any trauma or use of anticoagulation. He looked pale and in discomfort due to his pain.

**Figure 1.**
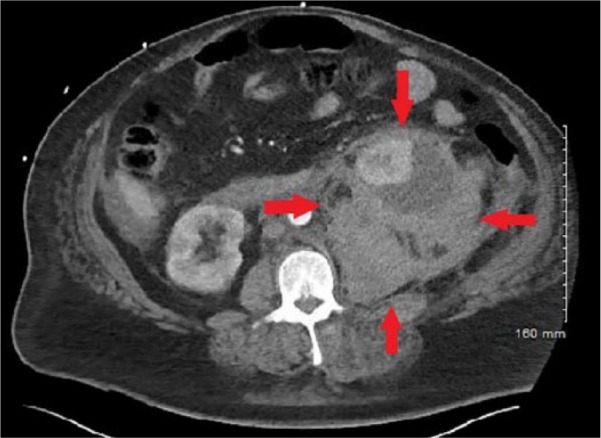
Computed tomography angiography scan of abdomen (axial view) showing retroperitoneal hematoma in the left suprarenal fossa causing mass effect on the left renal parenchyma.

**Figure 2.**
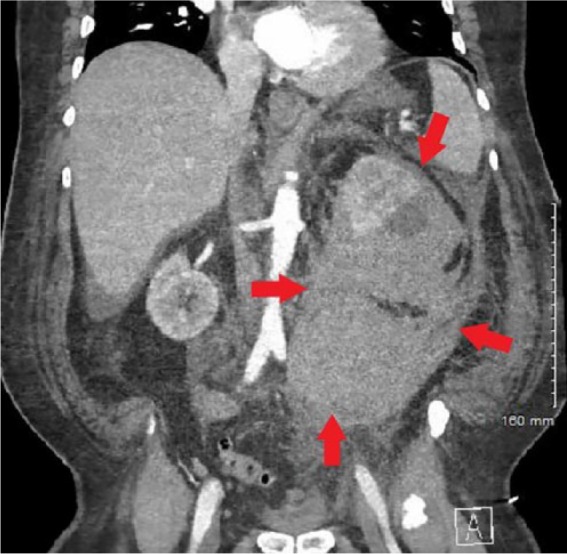
Computed tomography angiography scan of abdomen (coronal) showing retroperitoneal hematoma in the left suprarenal fossa and retroperitoneum extending into the left hemipelvis.

His vital signs included temperature of 37.5°C, pulse 107 beats per minute, respiratory rate 18 breaths per minute, blood pressure 104/62 mm Hg, and oxygen saturation of 97% on room air. On examination, he had severe tenderness in his left flank. His laboratory data were significant for creatinine of 1.6 from baseline of 0.9, hemoglobin 9.7 from baseline of 13.8, and lactate of 4.5. Computed tomography (CT) scan of his abdomen and pelvis was obtained, which showed large acute retroperitoneal hematoma extending into the left suprarenal fossa and left hemi pelvis anteriorly displacing the left renal parenchyma (Figures 1 and 2).

**Figure 3.**
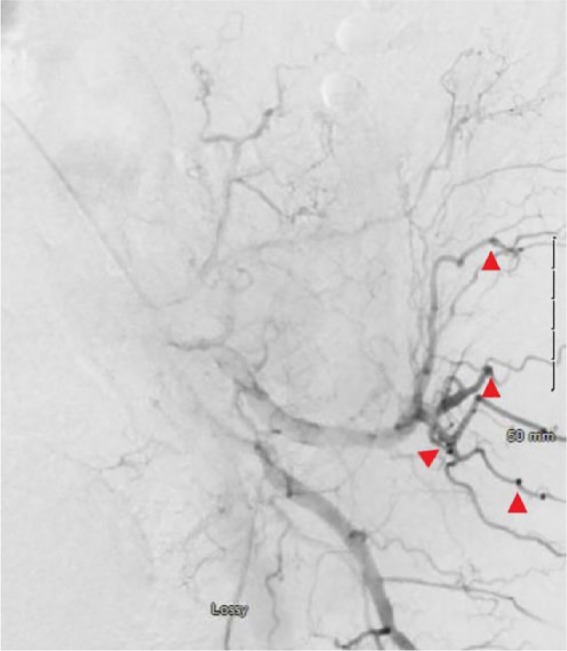
Left renal artery arteriogram showing multiple aneurysms.

**Figure 4.**
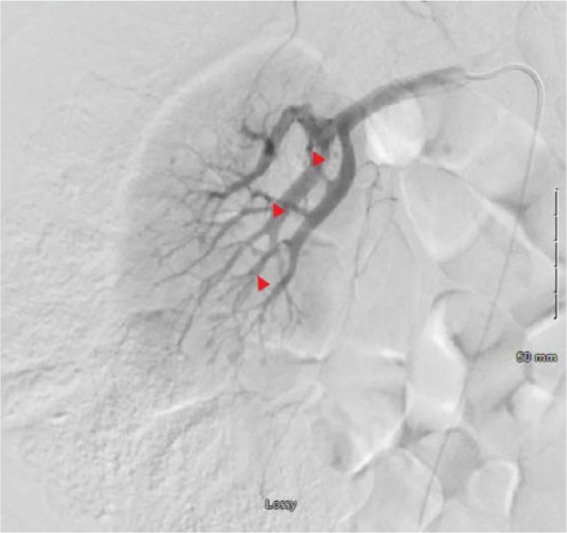
Right renal artery arteriogram showing multiple aneurysms.

His hemoglobin level decreased to 8.4 and he was transfused 2 units packed red blood cells. Interventional radiology was consulted who did an arteriogram that showed multiple bilateral renal artery aneurysms (Figures 3 and 4) and active extravasation present from L2 segmental artery trunk, findings consistent with polyarteritis nodosa (PAN), which was embolized that secured the bleeding, and the patient was started on prednisone 60 mg daily. Additional laboratory workup was obtained that showed elevated erythrocyte sedimentation rate >140, C-reactive protein 13.7, and low C4 with normal C3. ANA was positive with a titer of 1:80 with a nucleolar pattern. p-ANCA, c-ANCA, and hepatitis serologies were negative. His flank pain improved and hemoglobin remained stable. He was set up to see the outpatient rheumatology service and advised to continue prednisone and follow-up in 1 week.

## Discussion

Polyarteritis nodosa is a rare form of primary systemic vasculitis that is characterized by necrotizing inflammation of medium- or small-sized arteries with or with aneurysm formation^1^ and without involvement of arterioles, venules, and capillaries.^2^ The exact cause of PAN is unknown in the majority of the cases but it is found to be associated with hepatitis B, C, and human immunodeficiency viral infections.^1^

Signs and symptoms of PAN are primarily attributable to diffuse vascular inflammation and ischemia of the affected organs.^2^ It can affect any organ with the exception of lungs, with peripheral neuropathy and symptoms from osteoarticular, renal artery, and gastrointestinal tract involvement being the most frequent clinical manifestations.^2^

American College of Rheumatology has classified PAN on the basis of 10 criteria, which includes weight loss ≥4 kg, livedo reticularis, testicular pain or tenderness, myalgias, mononeuropathy or polyneuropathy, diastolic blood pressure >90 mm Hg, elevated blood urea nitrogen or serum creatinine levels, presence of hepatitis B reactants in serum, arteriographic abnormality, and presence of granulocyte or mixed leukocyte infiltrate in an arterial wall on biopsy. The presence of 3 or more of these 10 criteria was associated with a sensitivity of 82.2% and specificity of 86.6%.^3^

Spontaneous renal hemorrhage, also known as Wunderlich syndrome, is a rare but life-threatening condition characterized by acute, nontraumatic spontaneous bleeding from subcapsular and perirenal arteries leading to the formation of hematomas. A review by Zhang et al of 135 patients with spontaneous perirenal hemorrhage found that the most common cause was benign or malignant renal neoplasm (101, 61%) followed by vascular disease (28, 17%) with PAN occurring most frequently (20).^4^ Prompt diagnosis and treatment of Wunderlich syndrome is of paramount importance. Diagnosis is usually made by imaging studies including CT or magnetic resonance angiography^5^ and can be confirmed by arteriography, which characteristically shows multiple arterial aneurysms. Treatment is directed toward securing bleeding by selective embolization of the affected artery, which has shown promising results,^6^ and starting the patients on high-dose prednisone and/or immunosuppressive therapy. PAN associated with hepatitis or HIV infections is treated with antivirals and plasmapheresis.^7^

Our patient was found to have spontaneous renal hemorrhage that was diagnosed on CT angiography. He had 5 of 10 criteria for classification of PAN including weight loss, polyneuropathy, diastolic blood pressure >90 mm Hg, elevated creatinine, and arteriographic evidence of PAN. He underwent arteriography, which revealed underling multiple aneurysms in bilateral renal arteries consistent with PAN, followed by successful arterial embolization of the bleeding artery that secured the bleeding. Biopsy of renal arteries was not obtained due to concern for bleeding. He was started on high-dose prednisone for the treatment of PAN that resulted in improvements of his symptoms.

## Conclusion

Spontaneous retroperitoneal hemorrhage is a life-threatening condition that can be rarely caused by PAN. It should be considered in patients presenting with sudden-onset abdominal pain and anemia as it has a high morbidity and mortality if left undiagnosed and untreated.
